# The War in Ukraine, Agricultural Trade and Risks to Global Food Security

**DOI:** 10.1007/s10272-022-1052-7

**Published:** 2022-06-07

**Authors:** Thomas Glauben, Miranda Svanidze, Linde Götz, Sören Prehn, Tinoush Jamali Jaghdani, Ivan Đurić, Lena Kuhn

**Affiliations:** Leibniz Institute of Agricultural Development in Transition Economies (IAMO), Theodor-Lieser-Straße 2, 06120 Halle (Saale), Germany

**Keywords:** Q13, Q17, Q18

## Abstract

Any calls to move towards a centrally planned economy or autarky are strongly advised against, as this would only be to the detriment of food security in the Global South.

The war in Ukraine has aggravated existing tensions on the agricultural commodities market. Since late 2021, prices for commodities such as grains and vegetable oils have reached record highs, surpassing even the levels of the global food price crises of more than a decade ago. Now, the invasion of Russian forces in Ukraine has sent prices soaring even higher. This has above all affected import-dependent countries in the Middle East and North Africa (MENA) region and sub-Saharan Africa, which rely heavily on Russian and Ukrainian wheat. Disruptions to exports from the Black Sea region and high prices are further destabilising food security in these regions. However, global demand for wheat is expected to be met in the current marketing year since countries such as Australia, Brazil and the USA will increase exports to fill the gap left by Russia and Ukraine. It is difficult to predict what will happen beyond this marketing year, as this will be determined by the development of the current conflict in addition to agricultural fundamentals in key supply and demand regions. Global food systems and competitive international trade structures, in particular, are key to dealing with crises and mitigating the risks of food shortages. That way, disruptions in some exporting regions can be compensated for by exports from another. However, this requires greater collaboration in international trade. Any calls to move towards a centrally planned economy or autarky are strongly advised against, as this would only be to the detriment of food security in the Global South.

## Russia and Ukraine are key exporters of agricultural commodities

Russia is the top global exporter of wheat[1] and fertilisers, while Ukraine is the largest exporter of sunflower oil in the world and the fourth largest exporter of corn. Their combined export market share for 2015-2020 was 28% for wheat, 15% for corn, 66% for sunflower oil and 16% for fertilisers. In highly dynamic markets, Russia and Ukraine have almost tripled their export market share for wheat and sunflower oil over the past two decades while their combined export market share for corn has grown by a factor of seven. Fertiliser exports, on the other hand, have remained relatively stable (Figure 1).

The number of export markets has also increased, indicating a relatively high diversity of export structures. Between 2018 and 2020, 56 million tonnes of wheat and 31 million tonnes of corn were exported annually from Russia and Ukraine to 123 and 95 countries, respectively. The largest wheat export markets are Egypt (19%) and Turkey (13%), while the largest corn export markets are China (16%), the Netherlands and Spain (11% each), and Egypt (10%). Ten million tonnes of sunflower oil were shipped annually to 166 countries, with

1 Although Russia's export market share is considerable in some wheat-importing countries, empirical IAMO studies (e.g. Uhl et al., 2016; Pall et al., 2014) have yet to find any sign that Russian wheat traders influence prices on international wheat markets. Market structures can therefore largely be described as competitive rather than oligopolistic.

Figure 1

**Wheat, corn, sunflower oil and fertiliser exports on the world market**

Five-year average, 2001-2020

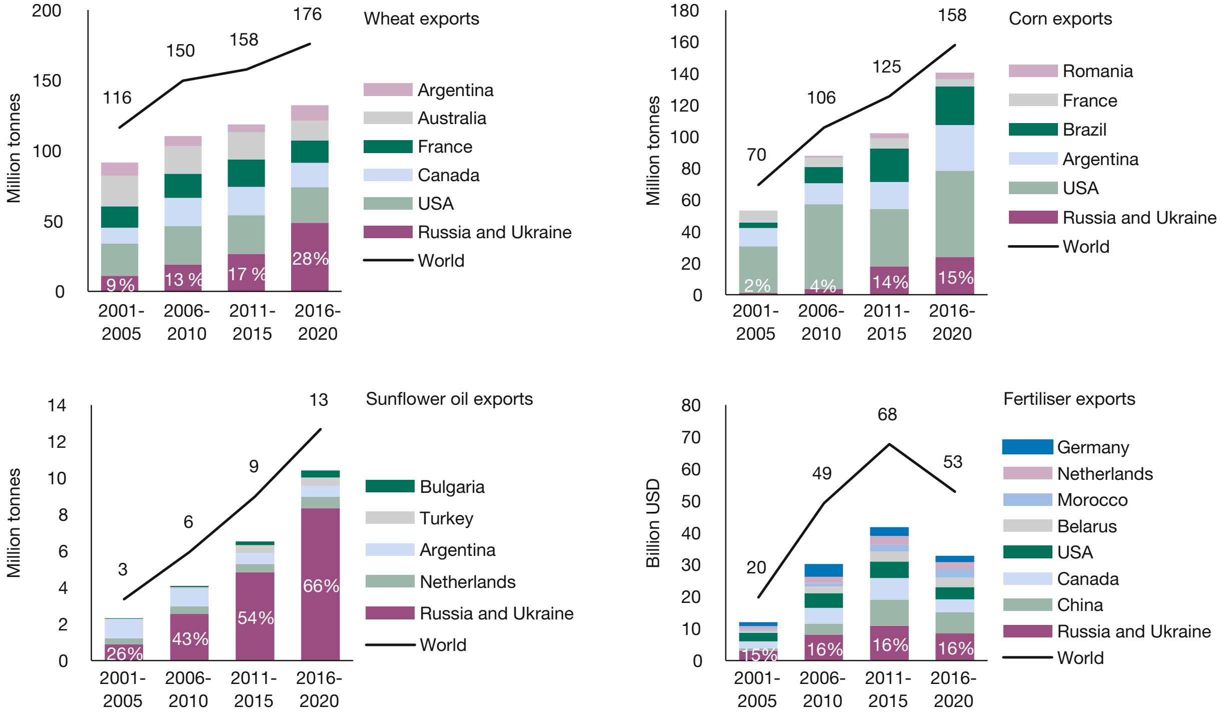

Source: UN Comtrade. Authors' representation.

the largest markets being India (27%) and China (15%). Mineral fertilisers from Russia and Ukraine were exported to 143 countries, with Brazil (21%), the USA (9%) and China (8%) as the main destinations. In countries in Africa, East Asia and the Pacific, demand for wheat imports rose rapidly, in particular for Russian wheat.[2] Meanwhile, the MENA region became the largest export market for Russian wheat (approximately 40% of Russian wheat exports).

## The MENA region benefits from Russian and Ukrainian wheat

Wheat is the main staple food for many of the world's poorer regions. The war in Ukraine is likely to have the greatest impact on regions that depend on imported wheat, particularly from Russia and Ukraine, as a key part of their diets. The greater this combination of factors, the more the population is at risk of suffering from food insecurity.

At highest risk are the 14 countries in the MENA region, the South Caucasus and Turkey, shown in Figure 2. The total combined population of these countries is around 330 million, and together they source more than 40% of their wheat from Russia and/or Ukraine. The most vulnerable are Albania, Egypt,[3] Lebanon, Libya, Georgia, Mauritania, Sudan, Tunisia and Yemen as large parts of their population are already subject to high risk of undernourishment (FAO et al., 2020).

Even countries that are less dependent on wheat imports from the Black Sea region could face food security issues. These include MENA countries such as Algeria, Morocco, Saudi Arabia and Jordan, as well as countries in Central Asia and Afghanistan, which consume large amounts of wheat per capita. Even though these countries import wheat mainly from regions other than Ukraine or Russia, (persistently) high wheat prices could have spillover effects for them. Furthermore, high wheat prices on world markets could also have a negative impact on less import-dependent poorer countries with high wheat consumption (such as Turkmenistan, Iran and Mongolia) if there is price transmission from the world to domestic markets.

2 Russian wheat exports to sub-Saharan Africa, East Asia and the Pacific rose from less than 5% in 2008-2010 to almost 30% in 2018-2020.

3 IAMO studies show how important Egypt is for global wheat markets. For example, Egyptian tender prices play a key role in price discovery on these markets. Furthermore, the price series of the three largest exporting countries, Russia, France and the USA, are highly integrated with Egyptian tender prices (see Heigermoser et al., 2021).

Figure 2

### Countries at "critical high risk" of food insecurity

| Population (million) | |
|---|---|
| Egypt | 102.3 |
| Turkey | 84.3 |
| Sudan | 43.8 |
| Yemen | 29.8 |
| Tunisia | 11.8 |
| Azerbaijan | 10.1 |
| UAE | 9.9 |
| Israel | 9.2 |
| Libya | 6.8 |
| Lebanon | 6.8 |
| Mauritania | 4.6 |
| Armenia | 3.0 |
| Georgia | 3.7 |
| Albania | 2.8 |
| Total | 328.9 |

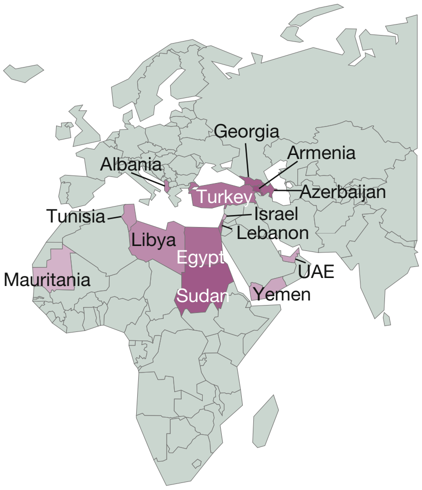

Share of imports from Russia and Ukraine in a country's wheat imports

41% 98%

Note: Wheat accounts for more than 20% of total per capita calorie intake (2019), import dependence accounts for more than 30% (2018–2019), and imports from Russia and Ukraine account for more than 30% (2018–2020).

Source: FAOSTAT, UN Comtrade, World Bank. Authors' representation.

## Continuously high prices in demand-driven global agricultural markets

As in previous years, global markets are demand-driven and prices remain high, particularly for wheat and vegetable oils.

At the beginning of 2021, the Food and Agriculture Organization (FAO) Food Price Index, which tracks monthly changes in prices, began to exhibit significant increases over previous years and, in January 2022, reached an all-time high (Figure 3). Similar trends were recorded for cereals and vegetable oils. For example, in January 2022, cereal prices rose by 33% while vegetable oil prices jumped by 80% from January 2020. In March 2022, these both rose a further 33% from January 2022, causing the Cereal Price Index to reach its highest level ever, exceeding the record prices of 2007/08 and 2010/11.

Euronext futures prices provide insight into price movements between the end of February and March 2022 as well as price expectations for the coming 2022/23 marketing year (ZMP, 2022).[4] Wheat futures quickly rose by 25% from

4 It is important to note that price increases appear more significant than they really are, as prices were converted from US dollars into euros and the euro lost value in 2022.

Figure 3

### Food and Agriculture Organization price indices

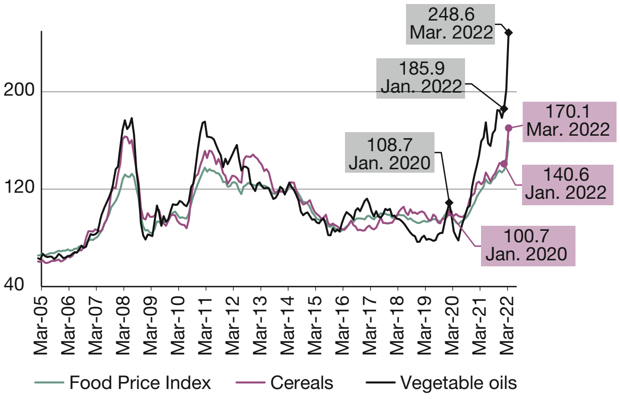

Source: FAO. Authors' representation.

€316.5/t (24 February, 2022) to a high of €396.5/t (7 March 2022). They have since fallen some 6% to €372.7/t (8 April 2022). The September futures contract (contract for the next harvest) is currently trading at around €352/t and the December contract is currently at €345/t (8 April 2022). The price of the corn futures contract has also risen 25% from €280/t (24 February 2022) to €351.5/t (7 March 2022) and has since been trading at a slightly lower level at around €320/t (8 April 2022) as well.[5] The November contract (next harvest) is currently trading at around €300/t. This indicates that the grain markets have somewhat calmed following initial panic, but remain at a high level[6] and are once again more strongly oriented towards (expected) fundamentals.[7]

Fertiliser prices also rose sharply between February and March 2022. The fertiliser price index rose by 43% from around 890 (25 February 2022) to 1270 (25 March 2022), possibly as a result of Russia's announcement of tempo-

5 Corn prices have risen again, most likely as a result of the US government's recent decision to increase the blending requirement of bioethanol in gasoline from 10% to 15%.

6 Further in-depth analysis would be needed to understand why grain prices remain at a relatively high level. However, it is very likely a symptom of the continuing uncertainty caused by the Black Sea conflict, ongoing supply chain disruptions due to the COVID-19 pandemic, growing import demand in China and in Africa, higher costs for inputs and rising crude oil prices. The latter, however, appear to have peaked on 7 March 2022 (approximately $123) and are now at levels similar to late January/early February 2022 (approximately $95; 7 April 2022) (Oil Price, 2022).

7 IAMO studies show that there have also been noticeable price reactions on the Chicago Board of Trade (increased price volatility) resulting from reports out of the Black Sea region, such as announcements of grain export restrictions in Russia. However, these prices were relatively quick to return to normal levels (see Heigermoser, 2022).

Figure 4

**Russian and Ukrainian wheat and corn exports: Observed (2006/07-2020/21) and forecast (2021/22)**

in million tonnes

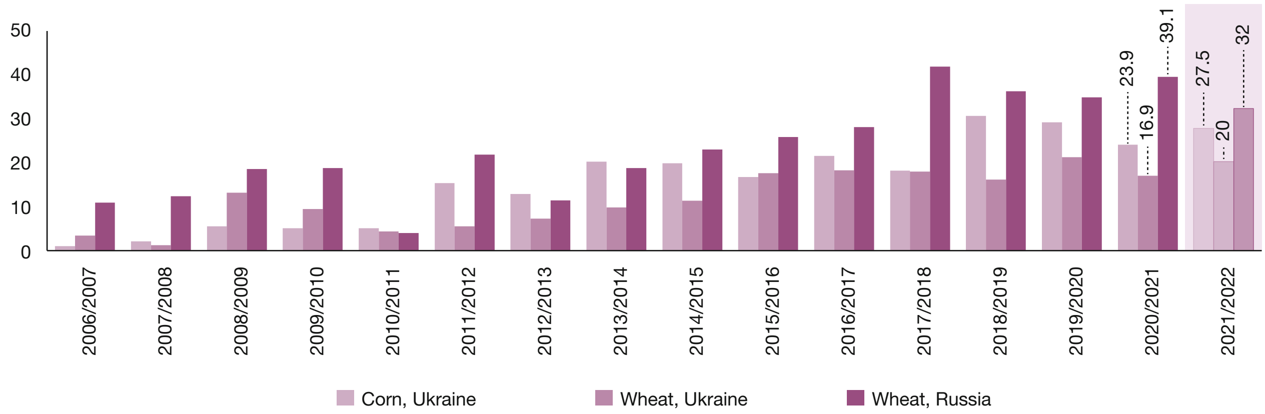

Source: USDA. Authors' representation.

rary export restrictions on fertiliser.[8] However, it should be noted that fertiliser prices had been rising since 2020/21 and at the end of 2021 (26 November 2021), the index was at 1,118 points, which is not far below the current level.

## Impact of grain shortages

Despite tensions on the export market, no physical shortages are expected in terms of global wheat supply. Furthermore, import destinations are mostly not expected to face shortages.

Russia has largely resumed exports via the Black Sea (Reuters, 2022a). However, as a result of sanctions, the US Department of Agriculture (USDA, 2022a) projects Russian wheat exports to fall by 8.6% (three million tonnes) below original forecasts for the 2021/22 marketing year. Financing restrictions and increased marine cargo insurance requirements are furthermore affecting shipments (Farm Futures, 2022a). However, agricultural products are exempt from the latest round of sanctions announced by the EU Commission banning transportation through EU territory and access to EU ports. Accordingly, Russian wheat shipments are expected to be around 32 million tonnes, which is slightly lower than export volumes in the 2018/19 and 2019/20 marketing years, but still higher than most export volumes over the past 15 years.

Currently, Ukrainian corn and wheat cannot be shipped via the Black Sea. Although efforts are underway to increase exports via rail and/or trucks travelling across the country's western borders, total volumes are likely to be very low, substantially due to the significant logistic challenges. Accordingly, the USDA has revised its original forecasts for Ukrainian corn and wheat exports in 2021/22 from 33.5 to 27.5 million tonnes for corn (down by 18%) and 24 to 20 million tonnes for wheat (down by 12%). Nevertheless, export volumes this marketing year are expected to be among the highest over the past 15 years (Figure 4).

Additional exports from Australia, Brazil, the EU and the USA are expected to fill the supply gap left by Russia and Ukraine this season. Contrary to this, India, intending to increase wheat supplies to the world market after the beginning of the war, has suspended private exports of wheat on 13 May 2022 due to the worsened harvest forecast. Despite this ban, Indian government still allows private exports contracted prior to the ban and considers future exports to foreign governments requesting supplies to meet their food security needs. For example, after the ban, India shipped 61,500 tonnes of wheat to Egypt and has received requests from governments of other countries (Haq, 2022). In addition, government officials from Egypt are holding "grain talks" with Argentina, France and the USA to offset potential shortages (Reuters, 2022b). Furthermore, the ENSO Outlook (2022) predicts that the unfavourable weather conditions caused by La Niña will dissipate over major growing areas in the USA and Europe. FranceAgriMer and the Deutsche Raiffeisenverband likewise

8 Russia imposed a two-month export ban on ammonium nitrate to control domestic prices on 8 February 2022. Nevertheless, Brazil imported about 900,000 tonnes of potash fertilisers from Russia since the beginning of the war. Furthermore, the permit procedures for exporting NPK fertilisers, which were introduced in December 2021, have been extended until the end of 2022. Russia's measures followed China's export ban on phosphate fertilisers, which is to last until June 2022. In addition, supply difficulties arose after several international shipping companies stopped loading at Russia's ports (Zinke, 2022). Russia is not currently expected to impose further supply restrictions, according to statements made by the Russian government on 5 April 2022.

Figure 5
**Global wheat and corn exports: Observed (2006/07-2020 /21) and forecast (2021/22)**
in million tonnes

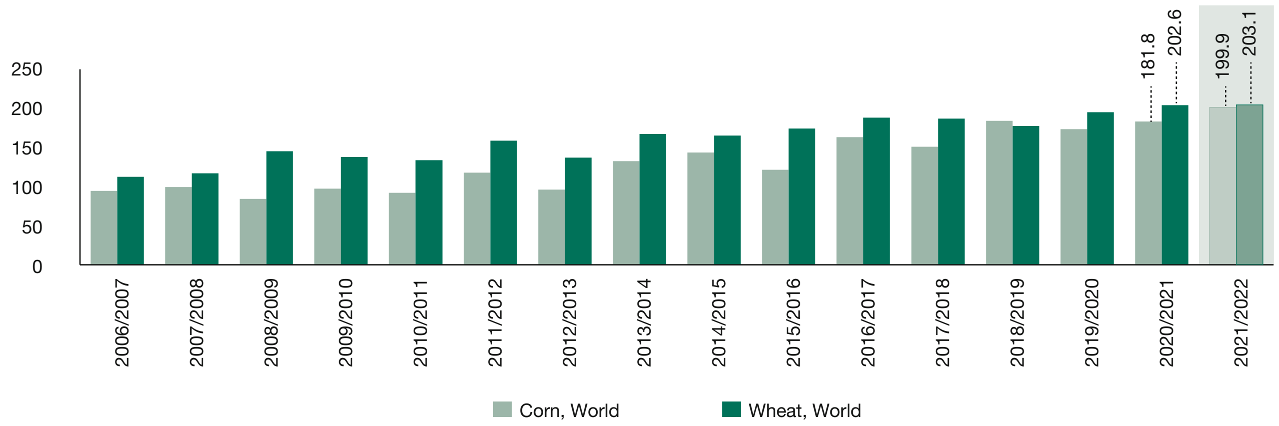

Source: USDA. Authors' representation.

do not predict any problems with wheat harvests in France and Germany, the two major wheat producers in Europe (Farm Futures, 2022b, 2022c).

The USDA expects only minor downward revisions to its original forecasts for total volumes of wheat and corn traded on the world market in 2021/22 (Figure 5). For both wheat and corn, this is (projected to be) around 200 million tonnes. As such, global wheat and corn trade would still be above the level of previous years.

### The situation remains critical for poorer, import-dependent regions

Even if no fundamental supply disruptions are expected on the world grain markets (so far) this marketing year, local supply gaps are likely to remain critical or possibly worsen as a result of the additional price increase in 2022, especially in the above-mentioned countries of the MENA region and in Africa.

In 2020, an estimated 118 million more people faced chronic hunger than in 2019 and 161 million more people experienced acute food insecurity, largely as a result of the COVID-19 pandemic. Overall, 320 million more people lacked access to adequate food in 2020 (World Bank, 2022). FAO estimates that the global number of undernourished people could increase by eight to 13 million as a consequence of the war in Ukraine. Of these, some three million will be in sub-Saharan Africa and one million in the MENA region. However, it remains unclear to what extent these increases are the result of previous developments, such as ongoing supply chain disruptions caused by the COVID-19 pandemic (FAO et al., 2020), or a direct result of the Black Sea conflict.

## Supply disruptions could intensify in the medium term, causing further food instability in the Global South

No noticeable respite is expected for the coming 2022/23 marketing year. As stated above, the September futures contract for wheat on Euronext (contract for the next harvest) is currently at around €350/t and the December contract is at €345/t. This means that trader expectations and uncertainties regarding the conflict have already been priced in. Nevertheless, market developments in the coming marketing year and beyond are difficult to predict, leaving room for speculation only. The major unknown variable is how long the conflict will last and if, when and how peace will eventually be reached. This will largely determine production and investment opportunities, market access and trade logistics, and the political (economic) conditions in the agri-food sector, especially in Ukraine and Russia. The extent to which these two countries will be integrated into international agricultural commodity markets in the future will also play a decisive role, as well as their willingness (or ability) to contribute to "smooth" market operations, the stabilisation of international prices during high-price rallies and, ultimately, to global food security. A key factor for Ukraine is how quickly and extensively it can rebuild its production and logistics infrastructure and whether it will have access to the sea for trade. Russia's future participation in global agricultural trade is likely to be influenced among others by the extent of sanctions.

This will subsequently affect the extent and the speed with which other world regions adjust to the changes, both in terms of supply and demand, as well as international trade flows and agricultural commodity prices. Beyond this, agricultural trade and global food supplies will continue to be exposed to parallel developments and (potential) crises. These include the repercussions of the COVID-19 pandemic, which has been ongoing for two years now; the growing demand for imports of grains, vegetable oils and fertilisers, particularly in China; and finally, weather conditions in various regions of the world.

The next question is how key producers on international and regional agricultural commodity markets will react in high-price phases. Many short-term effects can be mitigated via adjustments, in particular production and trade diversions. However, during the food price crises of 2007/08 and 2010/11, major grain exporters, including Russia and Ukraine, noticeably restricted their wheat exports by imposing quotas or even export bans with the aim of stabilising domestic prices as much as possible and generating tax revenues. Supply on international markets was restricted, international prices rose, further increasing the strain on consumers, especially in import-dependent developing countries (Svanidze et al., 2019). The Russian government furthermore introduced export restrictions on grains in response to price spikes in 2020,[9] and the Ukrainian government restricted vegetable oil exports to stabilise domestic consumer prices (Heigermoser and Glauben, 2021; Svanidze et al., 2021). Similar trade barriers were also observed in other countries (Laborde and Mamun, 2022).

At present, it cannot be ruled out that Russia or other exporting countries will extend or even increase wheat export restrictions to stabilise domestic prices and/or generate tax revenues under the current – most likely persisting – high prices on world markets. However, a complete export ban like the one imposed in 2010/11 as a result of poor harvests in the country seems rather unlikely at present. In particular, in anticipation of continuing economic sanctions, an influx of export revenues is needed, especially since the crop outlook is good but there is limited domestic storage capacity (Nasdaq, 2022).

A complete export ban and the resulting supply shortages coupled with higher grain prices would mostly hurt import-dependent regions, for example in Africa and Southeast Asia, who still exhibit a rather neutral position towards the conflict, while major wheat suppliers in the EU and North America would benefit greatly. It therefore appears very unlikely that Russia would impose massive export restrictions to provoke food insecurity in the import-dependent Global South and trigger waves of refugees to Western Europe or the USA, as is sometimes reported in the media. This would not be a viable geopolitical strategy, as supply and demand adjustments in other regions would largely compensate for supply shortfalls in the medium term. In addition, experience from the political unrest of the Arab Spring shows that waves of refugees from MENA countries did not flow into Europe despite massive bread price increases in 2007-2011. In this respect, it is more likely that Russia will increasingly apply export quotas or export tariffs to ensure, that enough wheat is available on the domestic market to stabilise domestic prices, and at the same time sufficient quantities of grain can be exported.

Price spikes on international grain markets combined with (possible) supply restrictions by major players often trigger reactions from other exporting nations (Djuric et al., 2015; Götz et al., 2013, 2016). For example, the current panic on international grain markets spilled over to the domestic market in Serbia, one of the major grain suppliers in the Western Balkans, leading to an increase in domestic prices. In order to stabilise domestic prices, the Serbian government consequently imposed an export ban on grains and corn on 10 March and on refined sunflower oil on 17 March. Similar reactions were observed, for example, in Hungary and Kazakhstan.

China is a different story. Although China is largely self-sufficient in wheat, it nevertheless eased existing import barriers to Russian wheat as early as 24 February 2022, in order to be able to meet domestic demand through storage and price stabilisation. China's increased demand is also expected to lead to higher prices on international markets. China has been trying to strategically diversify its imports for some time now. High corn imports, which so far mostly originated from Ukraine, are likely to be supplemented from the USA. Similar developments can currently be observed for most strategically important agricultural raw materials. Here, too, further intensified trade relations with North and South America are expected.

Overall, as long as major grain suppliers do not disrupt markets by imposing strict export restrictions, the war in Ukraine, *ceteris paribus*, is not expected to have a major impact on the global trade volume, i.e. global supply and global demand for key agricultural commodities in the coming 2022/23 marketing year. However, international agricultural production and trade flows may have to reorganise, which might lead to higher costs of global agricultural trade flows. Prices are likely, *ceteris paribus*, to rise or remain high with consumers in developing countries in particular forced to bear the burden. For European agriculture and consumers, no major effects on food supply are expected in the medium term.

## Openness to global trade is needed to cope with the crisis

The current conflict exposed and exacerbated tensions on international agricultural commodity markets existing amid

9 In addition to the export quota, which was introduced in 2020 in response to the COVID-19 pandemic and extended in 2021 and 2022, Russia imposed an export tax in July 2021, which taxes export prices above $200/t at a rate of 70%. However, on 15 February 2022, a floating export tax was implemented: If the price is between $200 and $375, the old rule applies; if the price exceeds $375 ($400), the price difference above $375 ($400) is taxed at 80% (90%) (USDA, 2022b).

the COVID-19 pandemic. Import-dependent countries with low per capita incomes are particularly vulnerable to shocks occurring amid the war in Ukraine, which further increase their risk of food insecurity. To overcome the challenges of potential food shortages, agricultural markets must be internationally open and competitive, and global supply chain structures must be in place to facilitate global trade. This would result in more resilient food markets and help mitigate the risk of food shortages by compensating for supply disruptions in one region with supply adjustments from another.

The smooth flow of goods across international borders is key to achieving and maintaining global food security, even in times of crisis. It is therefore advisable in the short term to reduce bureaucratic and tariff barriers to trade. An example of this is the Green Corridor, established in 2020 as a response to the COVID-19 pandemic, which facilitated cross-border trade between Western Balkan countries including Serbia, North Macedonia and Albania. Likewise, international business relations should be further diversified, although this may come at a cost. Currently there is no reason to panic buy or increase export controls on world grain markets in the coming marketing year, as markets appear to be calming. In addition, pressure should not be placed on import-dependent countries to stop wheat imports from specific regions, in particular Russia. Rather, targeted political efforts are needed to ensure that Ukraine and Russia remain integral parts of the world agricultural trading system. Their high production and export potential (Svanidze and Götz, 2019a, 2019b) remain important for combatting hunger in the Global South. This is especially true when global supply chain disruptions, such as those caused by the COVID-19 pandemic, or supply risks from other regions of the world endanger the food security of growing populations in import-dependent countries.

Last but not least, the current crisis must not be used as an excuse to once again bring about further large-scale reform of the European or global agricultural system – of any kind. While health and environmental aspects have to be part of agricultural production systems and supply chains, the planned-economy nature of the EU taxonomy as part of the European Green Deal is not the way to go about it. This will only lead to a shortage economy and invalidate achievements of market-oriented food systems of the past decades. Calls for ad hoc transitions to (more) closed food economies in the name of food security are likewise not advisable, as this would remove players from international markets, potentially lead to food shortages in many countries and take focus away from environmental and health-related issues. Instead, (unbureaucratic) actions are necessary to facilitate adaptation, innovation and resource-efficient processes along globally integrated agricultural production and supply chains, and ultimately promote growth and international trade.
